# A Mild Phenotype Caused by Two Novel Compound Heterozygous Mutations in *CEP290*

**DOI:** 10.3390/genes11111240

**Published:** 2020-10-22

**Authors:** Agnieszka Rafalska, Anna M. Tracewska, Anna Turno-Kręcicka, Milena J. Szafraniec, Marta Misiuk-Hojło

**Affiliations:** 1 Department of Ophthalmology, Wroclaw Medical University, 50-556 Wroclaw, Poland; anna.turno-krecicka@umed.wroc.pl (A.T.-K.); marta.misiuk-hojlo@umed.wroc.pl (M.M.-H.); 2 Łukasiewicz Research Network–PORT Polish Center for Research Technology, 54-066 Wroclaw, Poland; anna@datana.solutions (A.M.T.); Milena.Szafraniec@port.org.pl (M.J.S.)

**Keywords:** *CEP290*, retinitis pigmentosa, ciliopathies

## Abstract

*CEP290* is a ciliary gene frequently mutated in ciliopathies, resulting in a broad range of phenotypes, ranging from isolated inherited retinal disorders (IRDs) to severe or lethal syndromes with multisystemic involvement. Patients with non-syndromic *CEP290-*linked disease experience profound and early vision loss due to cone-rod dystrophy, as in Leber congenital amaurosis. In this case report, we describe two novel loss-of-function heterozygous alterations in the *CEP290* gene, discovered in a patient suffering from retinitis pigmentosa using massive parallel sequencing of a molecular inversion probes library constructed for 108 genes involved in IRDs. A milder phenotype than expected was found in the individual, which serves to prove that some *CEP290*-associated disorders may display preserved cone function.

## 1. Introduction

CEP290 is a centrosomal protein essential for proper ciliary function and, hence, crucial for cellular transport, signalling, organogenesis, and maintaining homeostasis. A wide spectrum of phenotypes is associated with *CEP290* mutations, ranging from isolated early-onset retinal degeneration (Leber congenital amaurosis—LCA (MIM 611755)), oculorenal dysplasia (Senior–Loken syndrome—SLNS (MIM 610189)), brain malformations (Joubert syndrome—JBTS (MIM 610188)) to lethal, multiple congenital anomalies (Meckel–Gruber syndrome—MKS (MIM 611134)). However, the underlying mechanisms for the heterogenous clinical presentation of *CEP290*-associated ciliopathies are not well elucidated, and the typical signs of these particular diseases may overlap [1].

In this paper, we report two novel *CEP290* variants, c.250+2T>C in intron 4 and c.7027del, p.(Val2343Phefs*4) in exon 51, discovered in a 30-year-old woman suffering from retinitis pigmentosa (RP). Although biallelic *CEP290* mutations are expected to result in congenital blindness or profound vision loss and nystagmus in early infancy [2], the patient presented with a mild phenotype of a non-syndromic retinal dystrophy with a strikingly well-preserved cone function after reaching adulthood. Our findings expand the phenotypic spectrum of *CEP290*-related ciliopathies to the mild end.

## 2. Materials and Methods

This study was approved by the local Bioethics Committee (approval no. 596/2016). Informed consent adhering to the tenets of the Declaration of Helsinki was received from the patient and her participating family members, and a Data Processing Agreement with a clause according to the General Data Protection Regulation EU Act was obtained.

The variants were identified as a part of a larger study of Polish patients suffering from inherited retinal disorders, and the methodology is described elsewhere [3]. Briefly, the patient’s whole blood was drawn using a BD EDTA tube (Becton-Dickinson, Franklin Lakes, NJ, USA) and PAXgene Blood RNA Tube (Qiagen, Hilden, Germany). Genomic DNA was isolated using QIASymphony DSP DNA Mini kit, and total RNA was isolated with QIASymphony RNA Kit on a QIASymphony robot (Qiagen). A NanoDrop 3300 spectrofluorimeter with Quant-iT™ dsDNA Assay Kit, broad range (Thermo Fisher Scientific, Waltham, MA, USA), served to precisely determine DNA concentration. The material from family members was self-collected using buccal swabs, and the DNA was isolated with a QIASymphony DNA Investigator kit (Qiagen).

Subsequently, we screened the sample using the molecular inversion probes (MIPs) method. The probes were designed in the Department of Human Genetics, Radboud University Nijmegen Medical Center, as previously described [4]. A total of 108 genes involved in the pathogenesis of IRDs were targeted in this panel, with the use of over 6000 MIPs covering the regions of interest. The libraries were prepared as previously described. The pools were then paired-end sequenced with a Rapid Run Mode on a HiSeq 1500 (2 × 100 cycles) (Illumina, San Diego, CA, USA), according to the manufacturer’s protocol. Afterwards, the samples underwent secondary analysis to create FASTQ files, which were then analysed using SeqNext module of the SeqPilot software (JSI medical systems, Ettenheim, Germany), using previously described filtering steps [3].

The novel consensus splice site variant was scrutinised using four in silico prediction programs, which are incorporated into AlaMut Visual Splicing Effects after module SpliceSiteFinder-like (SSFL), MaxEntScan (MES), NNSPLICE, GeneSplicer (Interactive Biosoftware, Rouen, France).

## 3. Results

### 3.1. Genetic Analysis

**Figure 1:**
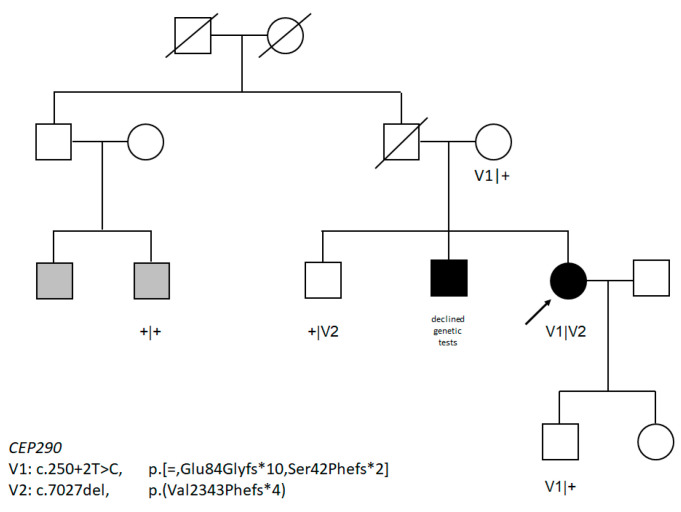
Pedigree of the family of affected proband (marked with an arrow). Variants are marked with V; + represents wild type allele.

The analysis of 108 retinal dystrophy genes yielded two novel *CEP290* variants, c.250+2T>C in intron 4 and c.7027del, p.(Val2343Phefs*4) in exon 51, which segregated within the family. One of the affected cousins, who also suffers from retinitis pigmentosa, did not have either mutation (Figure 1).

The variant c.250+2T>C, according to most utilised prediction programs, abolishes a consensus donor splice site (Figure S1, Table S1). According to in silico splicing modules present in AlaMut, it was predicted to cause a 28 bp nucleotide shift downstream of the donor splice site. This transcript, r.250_251ins250+1_250+28, was indeed detected and the activated cryptic splice site results in a premature stop codon further downstream the inserted sequence (p.(Glu84Glyfs*10)).

**Figure 2:**
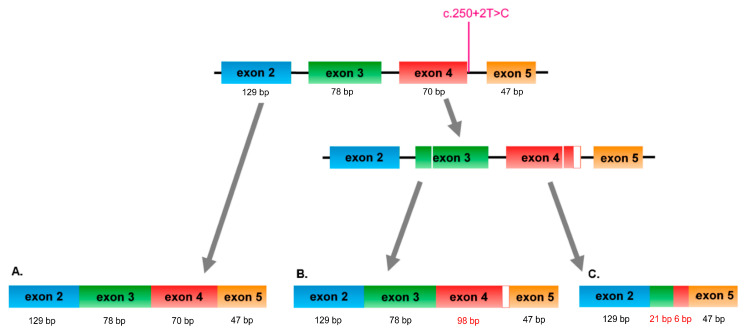
Splicing variants discovered in the proband. **A**. Normal transcript probably produced from both wild-type and c.250+2T>C allele. **B**. Longer transcript due to alternative splice site usage r.250_251ins250+1_250+28, p.(Glu84Glyfs*10). **C**. Truncated transcript, r.124_245del, p.(Ser42Phefs* 2).

Upon cDNA analysis directly from blood leukocytes of the patient, we also revealed a different splicing event that did not match any predictions. A new acceptor splice site was used within exon 3, and the donor splice site was shifted further upstream exon 4, resulting in the RNA level change r.124_245del, which would result in stop codon 2 residues on (p.(Ser42Phefs * 2)). (Figure 2, Figure S2). Splicing alterations are usually not fully effective, so the normal transcript was probably also produced with c.250+2T>C allele (Figure S3, upper band). However, since cloning of the alleles and splice assays were not feasible, it is not possible to determine that with absolute certainty.

The second allele variant, c.7027del in exon 51, is predicted to cause premature stop codon, p.(Val2343Phefs*4). Other pathogenic mutation in the vicinity has been reported, such as nonsense alteration c.7048C>T, p.(Gln2350*), which proves that downstream parts encoding for C terminus are necessary to provide the full functionality of the protein and strengthen the hypothesis of c.7027del causality. This transcript, along with the previous frameshift transcripts resulting from c.250+2T>C, may undergo further nonsense-mediated RNA decay, but this option was not tested, as it was not possible to culture patients’ cells with cycloheximide.

### 3.2. Clinical Description

The proband was a 30-year-old woman who started noticing visual field defects from the age of five. At the age of eight, she was diagnosed with RP based on visual field constriction to the central 10 degrees and characteristic fundus changes in optic nerve pallor, arterial narrowing, and bone spicules in the peripheral retina. Her corrected distance visual acuity (CDVA) at time of diagnosis was 0.6 in the right eye (RE) and 0.7 in the left eye (LE). The patient had low hyperopia; no nystagmus was present. Her visual acuity did not worsen throughout her childhood despite progressive visual field constriction. At the age of fifteen, both of her eyes were operated on due to alternating divergent strabismus. At this point, she reached excellent vision of 0.9 in both eyes.

**Figure 3:**
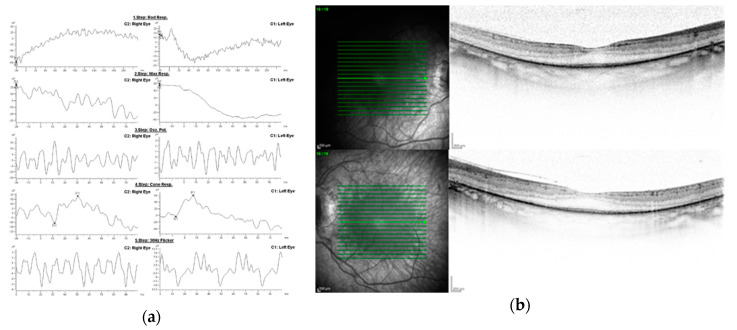
(**a**) Electroretinogram of the patient. (**b**) Macular optical coherence tomography and infrared image.

**Figure 4:**
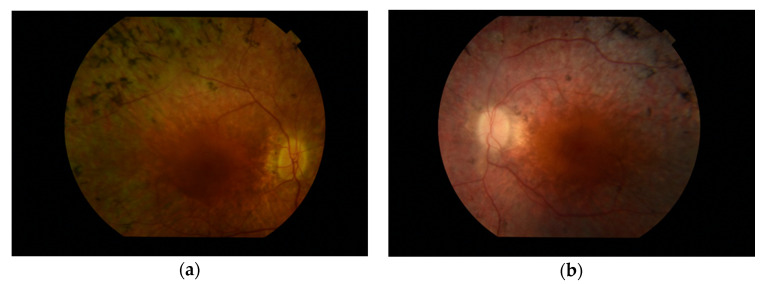
Fundus photographs of the right (**a**) and left (**b**) eye.

On presentation to our clinic at the age of 30, the patient had residual tunnel vision, CDVA of 0.6 in RE and 0.4 in LE, and full near visual acuity without vision aids (D-0.5). Her colour vision was unaffected. Optical coherence tomography (OCT) showed a well-preserved foveal anatomy with partial thinning of neurosensory retina in the macula and a normal peripapillary retinal nerve fibre layer thickness. Electroretinogram (ERG) revealed a negative scotopic and mixed response with photopic response decreased to 40 and 80 percent of the normal values in RE and LE, respectively (Figure 3). Fundus photographs are shown in Figure 4. 

The patient reported no systemic disorders. Magnetic resonance imaging of the brain and abdominal ultrasound were unremarkable.

Throughout her childhood, the patient received supportive treatment with multiple vitamins (B1, B3, B12, A, E) and intravenous pentoxifylline. As an adult, she regularly used alternative therapy with a variety of drugs with presumed antioxidant and neuroprotective properties, including cerebrolysine, solcoseryl, FiBS, idipacrine, Ginkgo biloba extract, and Aloe Vera extract.

## 4. Discussion

We report two novel *CEP290* variants, c.250+2T>C in intron 4 and c.7027del, p.(Val2343Phefs*4) in exon 51 in a woman suffering from RP. Although *CEP290* mutations are usually linked with severe ocular and systemic manifestations, the patient presented with an isolated retinal dystrophy with a relatively mild course despite early onset of symptoms. The two novel transcripts arising from the c.250+2T>C variant probably represent a minority of transcripts, since wild-type was present as a very strong band on an agarose gel in comparison to weak aberrant transcripts.

Two affected cousins from the paternal lineage also suffer from RP, although one of them, who was tested, did not carry the V2 mutation. This variant was probably present in patient’s paternal line, but since the father was deceased, his DNA was unavailable for testing. Most probably, the cousins represent a genocopy, which is a plausible event, especially given the enormous heterogeneity of retinal dystrophies and the estimated frequency of RP pathogenic alleles [5]. Unfortunately, we were unable to recruit proband’s affected brother, and nothing is known about his visual impairment except for the fact that he too was diagnosed with RP and has no systemic disease.

*CEP290*-related ciliopathies can be divided into syndromic (e.g., SLNS, JBTS, and MKS) and non-syndromic disorders. When not a part of a systemic disorder, *CEP290* mutations produce a phenotype of LCA, which is the earliest and most severe type of retinal dystrophy, resulting in blindness in the first year of life. The deep intronic mutation, c.2991+1655A>G, is by far the most common variant identified in large cohorts of patients suffering from IRD associated with *CEP290* mutations. Missense variants are rare. There is no clear genotype–phenotype correlation, and some studies showed contradictory results; patient homozygous for the most common intronic mutation had either better [6] or worse [7] final visual outcome. Perrault et al. studied 47 subjects belonging to 40 families segregating *CEP290* mutations and discovered that all patients displayed a severe cone-rod form of LCA with profound and early reduction in the visual function, along with high hyperopia and macular degeneration in the first decade of life [8]. *CEP290* variants have also been reported in a few patients with early onset severe retinal degeneration that was marginally different from LCA by the absence of nystagmus and oculodigital sign [9] or by a better preserved visual acuity and minimal measurable scotopic response to the ERG [10]. A retrospective analysis of 40 patients with mutations in *CEP290* recruited from the Moorfields Eye Hospital Inherited Eye Disease database found that all tested individuals had undetectable photopic ERGs consistent with a severe maculopathy despite relative preservation of foveal architecture on OCT imaging [11]. This cone-rich central island was seen independent of severity of visual loss, both in LCA patients and *CEP290*-mutant mice. A hypothesis for anatomically spared but poorly functioning central cones is that there is a putative intersegmental trafficking defect and that the cones remain viable despite impaired phototransduction efficiency [12].

Our patient retained a functional distance and near visual acuity at the age of 30, which along with just a moderately reduced photopic response in ERG, is indicative of relative cone sparing. The absence of cerebellar and renal involvement in our patient could be ascribed to the presence of a wild-type transcript, as hypothesized by den Hollander [13].

To the best of our knowledge, there have been three reports so far describing four cases of biallelic *CEP290* mutations causing isolated retinal dystrophy with preserved cone function after reaching adulthood. Ge et al. described a mild case of RP, with preserved full visual acuity at the age of 39, CEP290 heterozygous mutations p.(Glu1803Asp); p.(Phe1950Leufs*15) [14]. Birtel et al. reported the insertion of a cryptic exon and a premature stop codon in exon 12 and the most common deep-intronic mutation (c.2991+1655A>G) in intron 26 that created a splice-donor site. Another patient, heterozygous for c.982C>T, p.(Gln328*) and c.2991+1655A>G, p.Cys998* mutations, presented with RP causing vision loss from his twenties, and the final outcome was light perception at the age of 70 [15]. Two patients with biallelic *CEP290* mutations described by Barny et al. as having an unusually mild retinal disease in fact had macular involvement resulting in limited vision; one patient developed a chronic macular oedema refractory to treatment from the age of 10, and the other showed nystagmus and a poor visual acuity of 0.05 in RE and 0.2 in LE. Both had a non-recordable photopic response to the ERG [16].

In conclusion, the two novel *CEP290* mutations reported here cause a milder phenotype than expected, demonstrating that retinal disease due to *CEP290* mutations is not necessarily associated with poor cone function.

## Supplementary Materials

The following are available online at https://www.mdpi.com/2073-4425/11/11/1240/s1, Figure S1: In silico predictions for intron 4 splice site, Figure S2: cDNA sequence of *CEP290* transcript, Figure S3: Agarose gel electrophoresis, Table S1: In silico predictions for c.250T > C splice site alteration. References [17] are cited in the supplementary materials.
