# Supplementary material for: A Mild Phenotype Caused by Two Novel Compound Heterozygous Mutations in CEP290
Source: Genes (Basel). 2020 Oct 22;11(11):1240. doi: 10.3390/genes11111240 (PMC7690422; doi:10.3390/genes11111240)
Supplement: Supplementary file 1 [file genes-11-01240-s001.zip › Supplementary Data Captions.docx]

Supplementary Data Captions

Table S1: In silico predictions for c.250T>C splice site alteration.

Figure S1: AlaMut Visual display of in silico predictions for the intron 4 splice site.

Figure S2: cDNA sequence of *CEP290* transcript, Visible is the site where the deletion occurred due to the incorrect splicing, resulting in an aberrant, short transcript r.124_245del. Exon 3 is shown in green, exon 4 in red and exon 5 in orange. Potential novel donor splice site is marked pink, whereas potential acceptor splice site is marked in blue. The non-consensus cryptic splice site with AA dinucleotide might have been converted into AI via RNA editing – inosine is interpreted by cellular machineries (such as spliceosome) as guanosine, therefore a new AG splice site would be recognised

Figure S3: Agarose gel electrophoresis gel image. Lanes 1 and 8 (L) - size ladder (in base pairs); 2, 3, 4 - patient cDNA products; 5 - (C-) - negative control; 6 and 7 - wild type product from a healthy individual. In patient samples, visible are normal (400 bp) and mutated, longer (428 bp) products. The smaller product of ~280 bp, present in the patient, was not visible on gel.
