# Supplementary figures and images for: A Mild Phenotype Caused by Two Novel Compound Heterozygous Mutations in CEP290
Source: Genes (Basel). 2020 Oct 22;11(11):1240. doi: 10.3390/genes11111240 (PMC7690422; doi:10.3390/genes11111240)

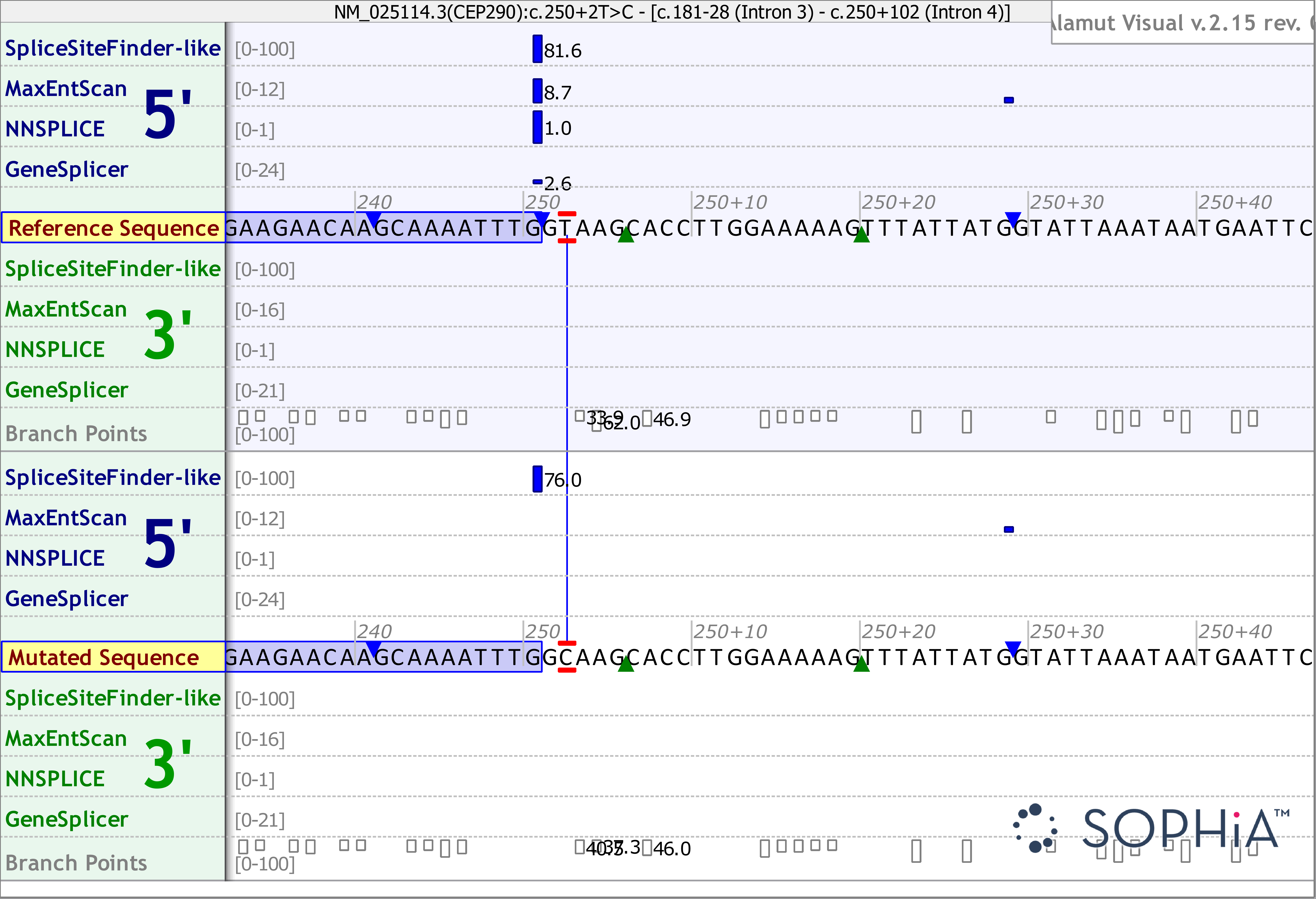

Supplement: Supplementary file 1 [file genes-11-01240-s001.zip › Supplementary Figure_1.tif]

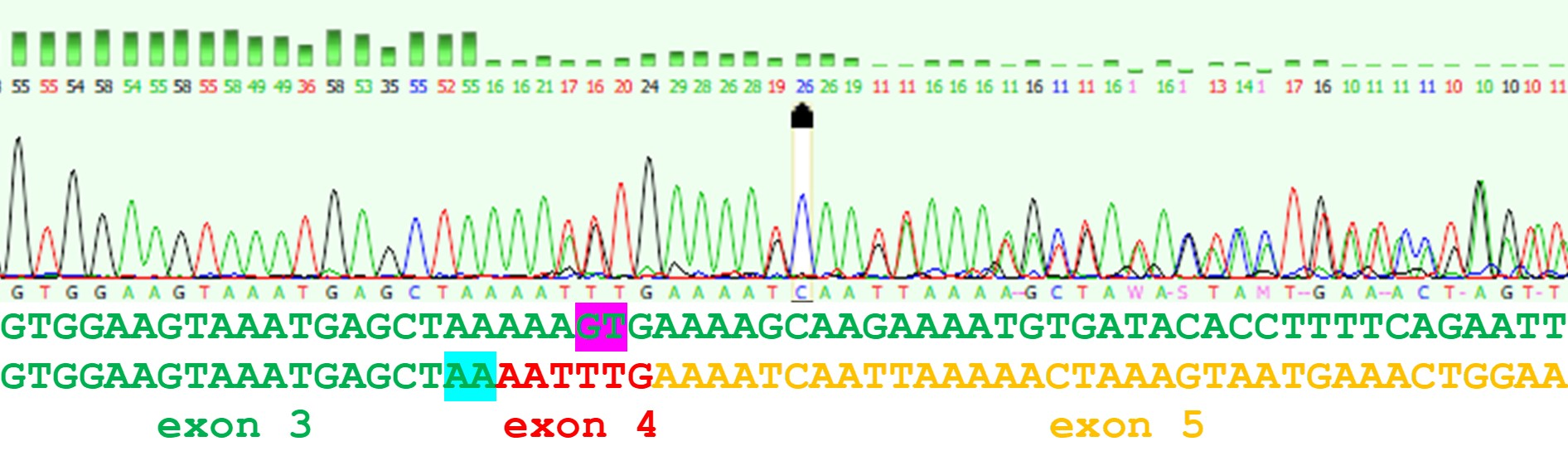

Supplement: Supplementary file 1 [file genes-11-01240-s001.zip › Supplementary Figure_2.tif]

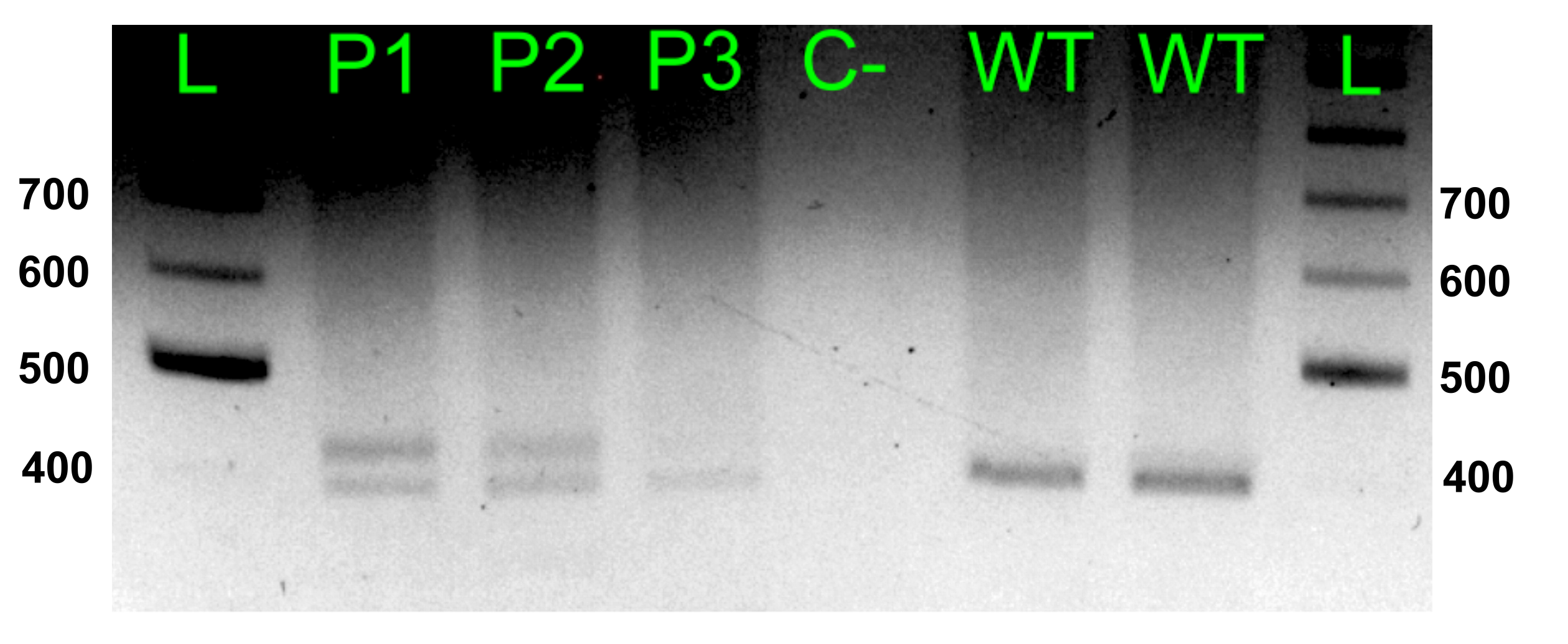

Supplement: Supplementary file 1 [file genes-11-01240-s001.zip › Supplementary Figure_3.tif]

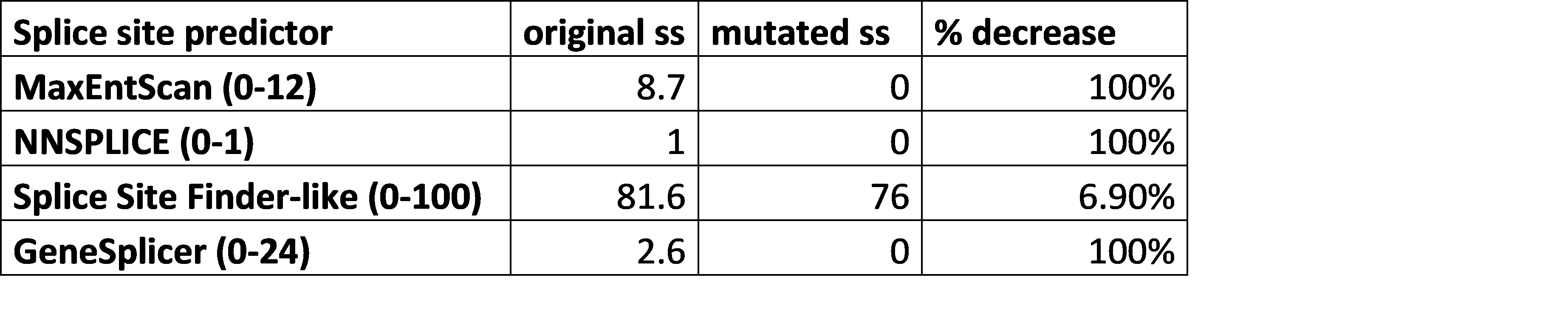

Supplement: Supplementary file 1 [file genes-11-01240-s001.zip › Table S1.tif]
